# Galectin-10 Characterization in Cleft Lip Palate – Affected Palatal Tissue

**DOI:** 10.15388/Amed.2025.32.1.3

**Published:** 2025-02-18

**Authors:** Alise Elizabete Rone, Mara Pilmane

**Affiliations:** 1 Riga Stradiņš University, Riga, Latvia

**Keywords:** galectin-10, cleft lip, cleft palate, galektinas-10, lūpos nesuaugimas, gomurio nesuaugimas

## Abstract

**Background:**

Cleft lip palate is one of the most common craniofacial birth defects in humans. Multiple defense factors have been described to have possible involvement in the failure in palatal shelve elevation, migration and fusion, most importantly, the role of chronic inflammation. A widespread presence of Gal-10 in different local inflammatory processes has been discussed before, however, knowledge of its involvement in local tissue inflammation in the postnatal cleft palate and tissue regeneration is scant. This study focuses on the detection of appearance and a possible role of Gal-10 in the cleft-affected facial tissue regarding its ontogenetical aspect.

**Materials and Methods:**

Craniofacial cleft tissue material was obtained from 21 children aged 8 months to 12.7 years undergoing veloplastic or uranoplastic procedure with non-syndromic craniofacial cleft diagnosis in milk or mixed dentition. Control groups for milk dentition were 5 subjects without orofacial defects for milk dentition and 3 subjects with plastic of superior lip frenula for mixed dentition. The number of factor positive cells in the control group and the patient group tissue was evaluated by using the semiquantitative counting method. The data were evaluated with the use of nonparametric statistical methods.

**Results:**

Elevated levels of Gal-10 were found in the epithelium in correlation with age, from milk to mixed dentition, as well as in both the control and the patient samples. Notable differences in expression can be seen by comparing the milk and the mixed dentition patient muscle tissue, where the milk dentition palate shows a more elevated factor expressed in comparison to the mixed dentition palate.

**Conclusions:**

The nearly total absence of Gal-10 in the healthy palate with an increase of its expression in the palatal epithelium from the milk to mixed dentition age of cleft-affected children suggests the possible role of this factor in providing the local defense function and the epithelium barrier function. The palatal muscles are not the main place for Gal-10 expression either in healthy or in cleft condition-affected individuals. The sporadic and insignificant appearance of Gal-10 only in the healthy milk dentition age and the mixed dentition age cleft-affected palatal connective tissue prove individual changes in the palatal tissue which does not depend on the specific disease.

## Abstract (it)

**Įvadas:**

Lūpos ir gomurio nesuaugimas yra vienas iš labiausiai paplitusių kraniofacialinių įgimtų defektų. Aprašyti keli protekciniai veiksniai, kurie gali turėti įtakos gomurio ar lūpos pakilimo, migracijos ir susiliejimo nepakankamumui, svarbiausias iš jų – lėtinis uždegimas. Plačiai paplitęs galektino-10 dalyvavimas įvairiuose vietiniuose uždegiminiuose procesuose buvo aptartas anksčiau, tačiau žinių apie jo dalyvavimą esant vietiniam audinių uždegimui, kai yra postnatalinis gomurio nesuaugimas ir audinių regeneracija, yra nedaug. Šiame tyrime daugiausia dėmesio skiriama galektino-10 atsiradimui ir galimam vaidmeniui veido audiniuose, kuriuos paveikė nesuaugimas, nustatyti ontogenetiniu aspektu.

**Medžiaga ir metodai:**

Kaukolės ir veido nesuaugimo audinių medžiaga buvo gauta iš 21 vaiko nuo 8 mėnesių iki 12,7 metų amžiaus, kuriems buvo atlikta veloplastinė arba uranoplastinė procedūra, kai buvo nustatyta nesindrominė kaukolės ir veido nesuaugimo diagnozė, esant pieniniam arba mišriam dantų sąkandžiui. Kontrolines grupes pieninio danties sąkandžio atveju sudarė 5 tiriamieji be orofacialinių defektų pieninio danties atveju ir 3 tiriamieji su viršutinės lūpos frenulės plastika mišraus danties atveju. Faktoriui teigiamų ląstelių skaičius kontrolinės grupės ir pacientų grupės audiniuose buvo įvertintas pusiau kiekybiniu skaičiavimo metodu. Duomenys vertinti neparametriniais statistiniais metodais.

**Rezultatai:**

Nustatyta, kad padidėjęs galektino-10 kiekis epitelyje priklauso nuo amžiaus, pieninio ir mišraus danties atveju tiek kontroliniuose, tiek pacientų mėginiuose. Ryškių raiškos skirtumų pastebima lyginant pieninio ir mišraus dantų sąkandžio pacientų raumeninį audinį, kur pieninio dantų sąkandžio gomuryje matoma padidėjusi veiksnio raiška, palyginti su mišraus dantų sąkandžio gomuriu.

**Išvados:**

Tai, kad beveik nėra galektino-10 sveikame gomuryje ir padidėjusi jo raiška gomurio epitelyje nuo pieninio iki mišraus danties amžiaus vaikų, turinčių nesuaugimą, atveju, rodo galimą šio veiksnio svarbą užtikrinant vietinę protekcinę funkciją ir epitelio barjerinę funkciją. Gomurio raumenys nėra pagrindinė galektino-10 raiškos vieta nei sveikų, nei nesuaugimo pažeistų asmenų organizme. Sporadiška ir nežymi galektino-10 raiška tik sveikų pieninio dantų amžiaus ir mišraus dantų amžiaus nesuaugimo pažeistame gomurio jungiamajame audinyje rodo individualius gomurio audinio pokyčius, kurie nepriklauso nuo konkrečios ligos.

## Introduction

*Cleft lip and palate* (CLP) is one of the most common craniofacial birth defects in humans affecting one in every 700 newborns [1]. In the 4^th^ gestational week, the development of the face begins with neural precursor cell migration developing a single frontonasal prominence, in two pairs of maxillary and mandibular processes. Throughout the 6^th^ and 7^th^ gestational weeks, the maxillary processes are fused first with lateral, followed by fusion with medial nasal processes, thus creating a primitive palate and upper lip [2,3].

Palatal shelves are formed form the maxillary processes, and this is followed by their shift from the vertical to the horizontal position. This is followed by midline fusion, initiated at the incisive foramen, and continued posteriorly. The secondary palate formation is initiated at the 6^th^ gestational week and continued into the 12^th^ week. Failure in the fusion of palatal shelves results in secondary cleft formation [34,35].

Failure of palatal shelf elevation, migration or fusion due to complex environmental and biological factors can lead to this birth defect [4]. It is one of the most prevalent craniofacial birth defects according to various literature, with multifactorial aetiology including genetic and environmental factors [13]. While the correlation between different biological tissue factors, such as Human beta defensin 2 to 4, interleukin 10, cathelicidin 37, has been proven to have a synergestic involvement in cleft palate formation, the role of Galectin-10 (Gal-10) still remains unclear [12].

Galectins (GAL) are a large family of proteins found in mammals that mediate the immune response by maintaining homeostasis and regulating immune functions [5, 6]. They have been proven to promote cell proliferation and apoptosis, but as they have also been found in patients with oncological diagnoses in different concentrations, more studies have been conducted to focus on isolating them as biomarkers for different pathologies [7,8]. Initially, galectins were found to be a family of 15 genes in which beta galactoside carbohydrates bind to galactose molecules, with one or two protein recognition domains [9,10,11]. Different research papers have focused on the multifunctional role of galectins in cellular stress responses, thus proposing that they may serve as biomarkers or mediators in this cellular stress [15,16].

Interestingly, galectin-10 has been found in eosinophils, and consequently in eosinophil-rich inflammation. This has been proven by finding *Charcot-Leyden crystals* (CLCs) – hexagonal pyramids varying in size [17]. They have been found to play an important role not only in inflammation and the immune response to this inflammation, but also in cell signaling, autophagy and migration [18,21].

While initially it was thought that Gal-10 could be found only in eosinophils, it has also been found in basophils, macrophages, and T-cells [24, 25]. Within the cell itself, it is located most commonly in the peripheral cytoplasm of eosinophils, in an area underneath the plasma membrane, where it is not stored in secretory granules. However, it can also be found in the nucleus, cytoplasm and the extracellular matrix [26,27]. Several eosinophilic diseases, based on this fact, have recognized galectin-10 as a potential and promising biomarker [19,22].

Despite the detection of Gal-10 in different eosinophilic diseases [30,31], virtually nothing is known about its appearance in cleft-affected tissue, thereby justifying the need for our study to detect its appearance as well as the possible role of Gal-10 in cleft-affected facial tissue from an ontogenetical aspect.

## Materials and Methods

### Material characteristics of subjects

This research was conducted in accordance with the 1975 Helsinki Declaration (revised in 2008) in Latvia at Riga Stradiņš University’s Institute of Anatomy and Anthropology.

The Ethical Committee of Riga Stradiņš University approved and independently reviewed this study (22 May 2003; 17 January 2013; 5/25 June 2018). After fully explaining the nature of the study, written informed consent was obtained from the patients’ parents for participation and publication of the obtained data. The material was obtained during veloplastic and uranoplastic surgery from patients diagnosed with partial uranoschisis, uranoschisis, or bilateral or partial (sinistral) cheilognathouranoschisis. Surgeries took place in the Cleft Lip and Palate Centre of the Institute of Stomatology of Riga Stradiņš University.

### Characteristics of tissue samples

The size of the obtained tissue sample was small, as the samples could only be obtained from the surplus of the tissue material that was not required for the surgery. If all of the tissue was needed for the closure of the palatal defect during the surgery, no samples could be taken. This concurs that the tissue sample could not be larger than 2 mm^2^.

### Characteristics of selected patients

The tissue material was obtained from 21 white children aged 8 months to 12.7 years.

For the milk dentition of thirteen patients, six were male and seven were female. Their age varied from 8 to 12 months, with an average of 9 months. The clinical diagnosis for these patients included a right-sided cleft lip palate (5 patients), a left-sided cleft lip palate (5 patients), and a bilateral cleft lip palate (3 patients).

In the mixed dentition group, there were eight patients, five males and three females. Their age ranged from 4.3 years to 12.7 years, with an average of 8.4 years. This group included 3 patients with a left-sided cleft lip palate, 1 patient with a bilateral cleft lip palate, 2 patients with a partial hard palate cleft, and 2 patients with a hard palate cleft (see Tables 1 and 2).

**Table 1:** Characteristics of cleft palate patients of milk dentition age

Patient number	Gender	Age (in months)	Clinical diagnosis
237	Male	8 months	Cheilognathouranoschisis sinistra
285	Female	8 months	Cheilognathouranoschisis dextra
319	Female	8 months	Cheilognathouranoschisis sinistra
335/1	Female	8 months	Cheilognathouranoschisis bilateralis
335/2	Female	8 months	Cheilognathouranoschisis bilateralis
261	Male	9 months	Cheilognathouranoschisis bilateralis
276	Male	9 months	Cheilognathouranoschisis bilateralis
326	Female	9 months	Cheilognathouranoschisis sinistra
332	Male	9 months	Cheilognathouranoschisis dextra
366	Male	9 months	Cheilognathouranoschisis sinistra
233	Male	10 months	Cheilognathouranoschisis dextra
298	Female	11 months	Cheilognathouranoschisis sinistra
17	Female	12 months	Cheilognathouranoschisis dextra
362	Female	12 months	Cheilognathouranoschisis dextra

**Table 2:** Characteristics of cleft palate patients of mixed dentition age

Patient number	Gender	Age (in years)	Clinical diagnosis
153	Male	4.3 years	Cheilognathouranoschisis sinistra
51	Male	5 years	Uranoschisis
2	Male	7.1 years	Uranoschisis partialis
242/1	Female	7.3 years	Cheilognathouranoschisis sinistra
18	Female	8.7 years	Uranoschisis
214/1	Male	11.1 years	Cheilognathouranoschisis bilateralis
5	Male	11.3 years	Uranoschisis partialis
121/2	Female	12.7 years	Cheilognathouranoschisis sinistra

### Sample selection criteria

#### Patient tissue

To select the patients and exclude as many co-factors as possible, inclusion and exclusion criteria were developed. The inclusion criteria were: 1) mixed dentition, 2) milk dentition, 3) both genders, 4) diagnosis of cleft lip or palate (Cheilognathouranoschisis sinistra, dextra, bilateralis, uranoschisis, uranoschisis partialis), 5) absence of another congenital disease, 6) indication for surgery, 7) no visible inflammation. However, the exclusion criteria consisted of: 1) age outside the mixed or milk dentition, 2) genetic syndromes, 3) chromosomal abnormalities, 4) immunodeficiencies, 5) visible inflammation.

Patient groups (during milk or mixed dentition) were selected as the subjects for this research. Because of the patients’ age, no major limitations arose. However, a potential limitation was a previous cleft lip surgery, as any viable tissue material is carefully used in the operation to close the orofacial defect gap in palates. While the tissue material was acquired over a 20-year period, due to the patients’ age and the specific procedure type, the availability of surplus tissue material was limited. As a result, only thirteen samples were available for milk dentition and eight for mixed dentition. No other major limitations for the tissue samples were established.

#### Control tissue

The control group tissue samples were collected from the Institute of Anatomy and Anthropology of Riga Stradiņš University; the samples came from the plastic of the superior lip frenula or necropsies performed post-mortem. The tissue material was obtained from eight children in both milk and mixed dentition, aged from newborn (28 days to 12 weeks) to 12 years, who did not suffer from any maxillofacial pathology (see Tables 3 and 4). Among the five patients with milk dentition, one was male, while four were females. Their age varied from newborn to 24 weeks. The clinical diagnoses for the causes of death included asphyxia by the umbilical cord, sudden death syndrome, or maternal health complications leading to abortion. Among the patients with mixed dentition, two were male, while one was female. Their ages ranged from 10 to 12 years.

The inclusion criteria for the control group were as follows: 1) absence of any maxillofacial pathology in the patient or the patient’s family history, and 2) lack of any other chromosomal or congenital abnormality, or inflammatory processes located in the oral cavity. Approval No. 2-PEK-4/595/2022 for the use of the control group tissue was issued on 14 December 2022.

**Table 3:** Characteristics of control cleft palate patients of milk dentition age

Patient number	Gender	Age (in years)	Clinical diagnosis
2b	Male	Newborn	Did not suffer any maxillofacial pathology
3b	Female	Newborn	Did not suffer any maxillofacial pathology
4b	Female	24 weeks old	Did not suffer any maxillofacial pathology
5b	Female	Newborn	Did not suffer any maxillofacial pathology
6b	Female	Newborn	Did not suffer any maxillofacial pathology

**Table 4:** Characteristics of control cleft palate patients of mixed dentition age.

Patient number	Gender	Age (In Years)	Clinical Diagnosis
3	Male	10–12 years	Did not suffer any maxillofacial pathology
8	Male	10–12 years	Did not suffer any maxillofacial pathology
10	Female	10–12 years	Did not suffer any maxillofacial pathology

All tissue samples from the examined control and patient groups were received with the permission and authorization of the parents of patients with the orofacial cleft and the deceased patients’ parents who donated their samples. The control and study groups were considered appropriate for comparison because they belong to the milk (6 months – 33 months) [20] or mixed (5 to 13 years) dentition age.

#### Routine morphological assessment

The fixation of the secured tissue material was performed within 24 hours by using 0.1 M phosphate buffer with a pH of 7.2, 2% formaldehyde, and 0.2% picric acid. The specimens were processed through a washing procedure using a phosphate buffer containing 10% saccharose and saline solution. Following this, the tissue was embedded in paraffin, and the specimens were cut into 5–7 µm thin sections by using a microtome. To assess the morphological structures of the soft palate, haematoxylin and eosin staining was performed.

#### Immunohistochemical analysis

To detect the amount of the galectin-10 defense factor in the selected tissue samples immunohistochemically, routine streptavidin and biotin immunostaining methods were used for the preparation of tissue samples. An antibody dilutant (code-938B-05, *Cell MarqueTM*, Rocklin, CA, USA) was applied to attain antibody dilution. Before and after washing the slide with the TRIS buffer, blockage with 3% peroxide solution was performed. This was followed by an hour-long incubation with the primary antibodies, and another washing with the TRIS buffer three times. HiDef DetectionTM reaction amplificator (code 954D-31, *Cell MarqueTM*, Rocklin, CA, USA) was used afterwards for 10 minutes, followed by another washing with the TRIS buffer. HiDef DetectionTM HRP Polymer Detector (code-954D-32, *Cell MarqueTM*, Rocklin, CA, USA) was used to perform incubation for 10 minutes, followed by triple washing with TRIS buffer solution. DAB+ chromogenic liquid DAB Substrate Kit (code 957D-60, *Cell MarqueTM*, Rocklin, CA, USA) was used to layer the slides for 10 minutes, followed by rinsing under running water, and stained with hematoxylin (code-05-M06002, Mayer’s Hematoxylin, *Bio Optica Milano S.p.A*., Milano, Italy) to contrast. Dehydration, which was increased from 70 to 90 degrees of ethanol, was used for the finishing steps of the immunohistochemical staining, continued by clarification with carboxylic acid. The slide was sealed with a coverslip.

Detection of galectin-10 (ab157475, working dilution 1:200, *Abcam*, Cambridge, UK) was performed.

#### Assessment of local tissue defense factor quantity

To assess the immunoreactive cell appearance and distribution of galectin-10 in the muscle, connective tissue or epithelium, semi-quantitative non-parametric tests and light microscopy were performed on the slides containing tissue samples. The slides were evaluated by two independent histologists. A scale, with labels summarized in Table 5, was used to grade structures stained positively within the visible field. A *Leica DC 300F* digital camera (*Leica Microsystems Digital Imaging*, Cambridge, UK) was used to take illustrative images of the stained tissue samples, followed by the use of the *Image Pro Plus* program (*Media Cybernetics, Inc*., Rockville, MD, USA) to analyze and process the pictures.

**Table 5:** Scale used in the semi-quantitative evaluation for galectin-10

Identifier used	Explanation
0	No positive structures in the visual field (0%)
0/+	Occasionally positive structures (12.5%)
+	Few positive structures (25%)
+/++	Few to moderate numbers of positive structures (37.5%)
++	Moderate number of positive structures (50%)
++/+++	Moderate to numerous numbers of positive structures (62.5%)
+++	Numerous numbers of positive structures (75%)
+++/++++	Numerous to abundant numbers of positive structures (87.5%)
++++	Abundant numbers of positive structures (100%)

### Statistical analysis

Evidence-based statistical analysis and methods borrowed from the biomedical research checklist (SAMBR) were used to perform data analysis. As previously mentioned, the positive cell count was used instead of their exact quantity. To measure the results, non-parametric tests were performed. Statistical analysis was carried out by using *SPSS* statistics, version *27.0* (*IBM Company*, Chicago, USA). For each statistical test, a p-value of <0.05 was required for the results to be considered statistically significant. This was achieved by using either descriptive or analytical statistics methods. Antibodies were counted by using the semi-quantitative evaluation; therefore, the data obtained from the tissue samples were ordinal. This further signified the need to use non-parametric tests to calculate the results.

#### Fisher-Freeman test

The Friedman’s two-way ANOVA test is a non-parametric test used to evaluate repeated measures analysis of variance. When the dependent variable is ordinal, it can be used to test differences between groups. Furthermore, it can be used when continuous data, typically used to run a one-way ANOVA with repeated measures, violates the fundamental inferences [28].

#### Kruskal-Wallis test

The Kruskal-Wallis test is a non-parametric test used to evaluate independent samples serving the objective to analyze the variance of expression of a neuromodulator between the patient group and the control group samples. When presented with an independent variable on a continuous or ordinal scale within two or more groups, it can be used to detect statistically significant differences [28].

#### Spearman’s rank correlation

To evaluate and reveal correlations between the indices of different groups, Spearman’s rank correlation was used. The following values of Spearman’s rho were used, where R <0.2 was assumed to represent a very weak correlation, R values of 0.20–0.39 indicated a weak correlation, R values from 0.40 to 0.59 indicated a moderate correlation, R values of 0.60–0.79 indicated a strong correlation, while R values above 0.80 indicated a very strong correlation.

Each statistical result for the tests to be valued as statistically significant needed to have a p-value <0.05 [28].

## Results

### Routine staining

**Figure 1:**
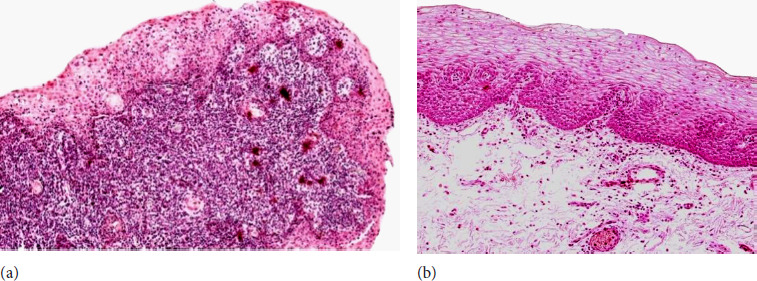
**(a** and **b)**. Haematoxylin and eosin staining of the control and patient tissue samples. **a)** Control sample with non-keratinized stratified squamous epithelium with epithelial vacuolization, and subepithelial inflammatory cell infiltration. 200x; **b)** patient sample with minor subepithelial cell infiltration and basal cell hyperplasia. 200x

The control group data obtained from healthy individuals consisted of mucosal connective tissue and non-keratinized stratified squamous epithelium (Figure 1a). Routine staining revealed epithelial vacuolization, subepithelial inflammatory infiltration and some basal cell hyperplasia (Figure 1 a,b).

### Galectin-10 in milk dentition age patients

**Figure 2:**
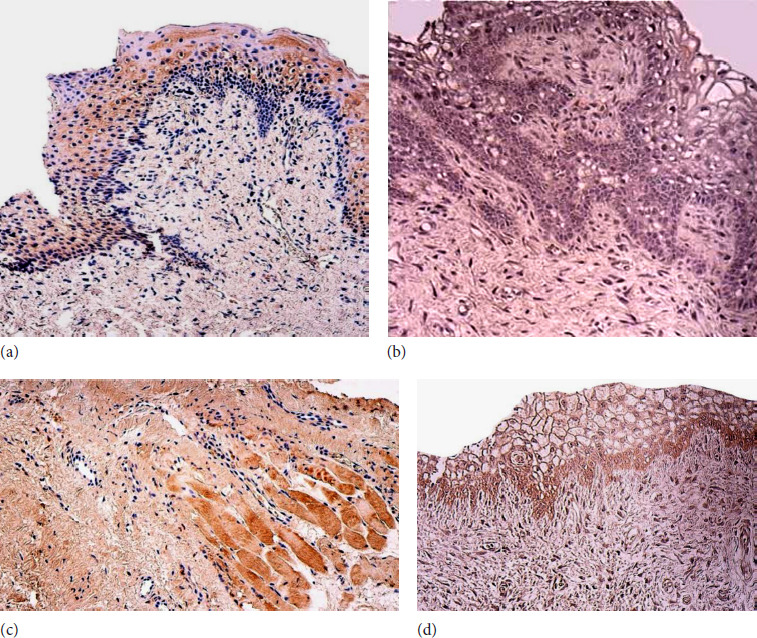
**(a–d)**. Soft palate in children with cleft palate and control group in milk dentition: **(a)** Moderate number of Gal-10 positive cells in the epithelium of patient. Gal-10 IMH, x200; **(b)** Lack of epithelial expression of Gal-10 in the control group. Gal-10 IMH, x200; **(c)** Moderate number of Gal-10 positive cells in the patient palatal muscle tissue. Gal-10 IMH, x200; **(d)** Moderate number of Gal-10 positive cells in the connective tissue in the control group. Gal-10 IMH, x200

The average amount of galectin-10-positive structures in the epithelium of the patient group was few (+), with variations from none to a moderate number. The average amount of positive structures of galectin-10 in the palatal muscle tissue of the patient group was few (+), with variations from none to numerous. The average amount of positive structures of galectin-10 in the connective tissue of the patient group was none, with variations from none to few positive structures (Figure 2 a,c; Table 6).

**Table 6:** Semi-quantitative evaluation of galectin-10 positive cells in patient cleft soft palate tissue in milk dentition

Patient number	Epithelium	Connective tissue	Muscle tissue
237	++	0	0
285	0/+	0	0/+
319	0/+	0	+++
335/1	+	0	0
335/2	++	0	0
261	++	0	0
276	0	0	++
326	+	0	++
332	++	+	0
366	++	0	++
233	0	0	++
298	0/+	0	++
17	0	0	++
362	0	0	+++
Median	+	0	+

Abbreviations: 0 – no positive structures, 0/+ – occasional positive structures, + – few positive structures, ++ – a moderate number of positive structures, +++ – numerous numbers of positive structures.

### Galectin-10 in milk dentition control

For the median value of galectin-10-positive structures in the control group, the epithelium revealed no positive structures, with fluctuation from none to a few positive structures at the median value. In contrast, for the connective tissue, the median number fluctuated from few to a moderate number of positive structures (+/++), with fluctuations ranging from none to moderate numbers. Additionally, no muscle tissue was found in the control group for comparisons (Figure 2b,d; Table 7).

**Table 7:** Semi-quantitative evaluation of galectin-10 positive cells in the milk dentition control group of cleft soft palate tissue

Patient number	Epithelium	Connective tissue	Muscle tissue
2b	0	+/++	0
3b	0	+	0
4b	0	0/+	0
5b	+	++	0
6b	0	0	0
Median	0	+/++	0

Abbreviations: 0 – no positive structures, 0/+ – occasional positive structures, + few positive structures, +/++ few to moderate numbers of positive structures, ++ moderate numbers of positive structures.

### Galectin-10 in mixed dentition age patients

**Figure 3:**
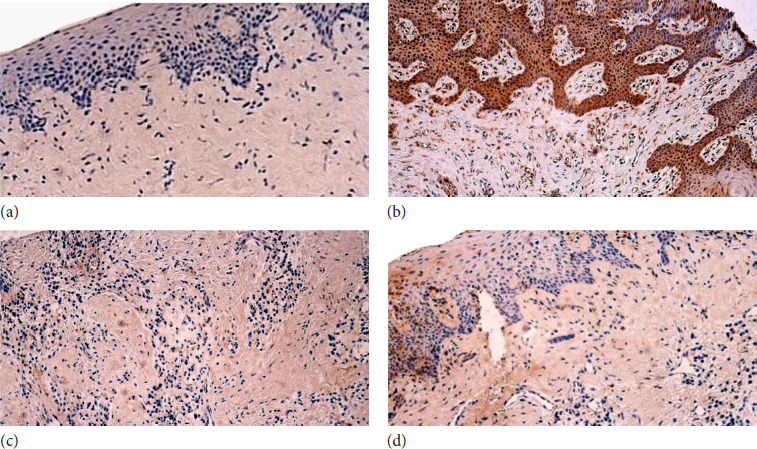
**(a–d)**. The soft palate in children with cleft palate and the control group in mixed dentition: **(a)** Lack of Gal-10 positive cells in in the connective tissue of a patient. Gal-10 IMH, x200; **(b)** Slide containing numerous Gal-10 positive structures in the connective tissue and epithelium of a patient. Gal-10 IMH, x200; **(c)** Control group patient’s connective tissue with absence of positive structures. Gal-10 IMH, x200; **(d)** Few to moderate numbers of positive structures in the epithelium of the control group. Gal-10 IMH, x200

For the median value of galectin-10-positive structures in the patient tissue sample, epithelium was few to moderate (+/++), with fluctuations from none to abundant. In contrast, the median quantity of galectin-10 in the connective tissue samples of the patient group showed few positive structures (+), whereas variations ranged from none to numerous. Furthermore, the median quantity of galectin-10 for the muscle tissue showed no positive structures, with variations ranging from none to numerous (Figure 3a,b; Table 8).

**Table 8:** Number of galectin-10-positive cells in the patient cleft soft palate tissue in the mixed dentition group

Patient number	Epithelium	Connective tissue	Muscle tissue
153	+	0	+
51	0	0	+++
2	+++	+	0
242/1	+/++	0	0
18	+++	+++	0
214/1	++	0	0
5	+++	+	0
121/2	0	++	+
Median	+/++	+	0

Abbreviations: 0 – no positive structures, + – few positive structures, +/++ – few to moderate numbers of positive structures, ++ – moderate numbers of positive structures, +++ – numerous numbers of positive structures.

### Galectin-10 in mixed dentition control

For the control group, the median value of galectin-10 found in the epithelium was none to few positive structures (0/+), with variations ranging from none to a moderate number to numerous. However, the median value for the connective tissue was no positive structures with variations ranging from none to a moderate number of positive structures, with not enough material for the muscle tissue found in the slides (Figure 3c,d; Table 9).

**Table 9:** Number of galectin-10 positive cells in the patient cleft soft palate tissue in the mixed dentition control group

Patient number	Epithelium	Connective tissue	Muscle tissue
3	0	0	0
8	0/+	++	0
10	+/++	0	0
Median	0/+	0	0

Abbreviations: 0 – no positive structures, 0/+ – occasional positive structures, +/++ – few to moderate numbers of positive structures, ++ – moderate numbers of positive structures

### Correlations in the epithelium, connective, and muscle tissue of the milk and mixed dentition age

For neither milk dentition with control and patient samples, nor mixed dentition patient control samples in the epithelium and connective tissue, were any statistically significant differences observed. Statistical differences were not revealed by the Fisher-Freeman test. For the connective tissue comparison between the milk dentition patient and the control group, the statistical correlation was p=0.119. The epithelium, however, revealed correlation levels of p=0.070. For the comparison of both the connective tissue and the epithelium in the mixed dentition between the mixed dentition and the control group, the correlation was p=0.357, but, in the muscle tissue, a value of p=1.00 was found. Statistically significant correlations of the tissue factor in tissue samples from the patient and control groups are presented in Table 10.

**Table 10:** Statistically significant correlation coefficient comparison analysis between Gal-10 in different tissue samples and groups

Probability of occurring by chance	Correlation between patient and control group	p-value
No	Gal-10 in Mlk dentition E and Mlk dentition control E	0.070
No	Gal-10 in Mlk dentition CT and Mlk dentition control CT	0.119
No	Gal-10 in Mx dentition E and Mx dentition control E	0.357
No	Gal-10 in Mx dentition CT and Mx dentition control CT	0.357
No	Gal-10 in Mx dentition M and Mx dentition control M	1.00

Abbreviations: Gal-10 – galectin-10, CT – connective tissue, E – epithelium, M – muscle tissue, Mx – mixed dentition, Mlk – milk dentition, Mx c – mixed dentition control, Mlk c – milk dentition control.

The median values for each tissue sample showed that galectin expression in the epithelium was the highest in mixed dentition, with a few to moderate numbers of positive structures, and the lowest in the milk dentition control group. For the connective tissue, the highest expression with few to moderate expression was in the milk dentition control, but the lowest with no positive structures in both the milk dentition and the mixed dentition control group. For the muscle tissue, the highest expression reached few positive structures in the milk dentition and no positive structures for the remaining groups. The complete set of the median values of the factor in the studied groups is shown in Table 11.

**Table 11:** Median value of immunohistochemical evaluation

Groups	Epithelium	Connective tissue	Muscle tissue
Milk dentition	+	0	+
Milk dentition control	0	+/++	0
Mixed dentition	+/++	+	0
Mixed dentition control	0/+	0	0

Abbreviations: 0 – no positive structures, + – few positive structures, +/++ – few to moderate numbers of positive structures, ++ – moderate numbers of positive structures, +++ – numerous numbers of positive structures.

## Discussion

A widespread presence of Gal-10 in different local inflammatory processes has been discussed before; however, knowledge of its involvement in the local tissue inflammation in postnatal cleft palate and tissue regeneration is limited. Until now, Gal-10 has been identified in eosinophilic inflammations, such as chronic rhinosinusitis, or esophagitis, but not in a cleft-affected tissue [23,33].

Patients with cleft lip and/or palate in the milk and mixed dentition age are required to undergo multiple operations. Therefore, much more prominent inflammation is created, which can further disrupt the tissue remodulation and healing. This is important when considering which local tissue defense factors could be involved, and whether these fluctuations from the innate tissue immune response could affect the tissue regeneration.

Our results covered the palatal tissue affected by cleft palate and/or lip, particularly epithelium, muscle, and the connective tissue as well as the control group tissue, in both milk and mixed dentition as the main places for Gal-10 expression as a local defense factor. We found elevated levels of Gal-10 in epithelium in correlation with age, from milk to mixed dentition, as well as in both control and patient samples. This tendency also refers to the elevated levels of Gal-10 in the epithelium of the mixed dentition group. This suggests that Gal-10 can act as a local epithelial defense factor as it is absent in the connective tissue, which is observed very clearly in the milk dentition group. This allows to further speculate that a parafunction of this defense mechanism could increase the potential risks of inflammation and a decrease in the healing aspect in the cleft-affected tissue. We could further hypothesize that the defense factor expression is upregulated by the maturation of the tissue.

Interestingly, in the Gal-10 expression of the muscle and the connective tissue, no differences were observed between the patient and the control group, which, furthermore, suggests that cleft lip and/or palate does not regulate its expression. Lack of significant galectin-10 expression in a cleft-affected connective tissue proposes that keratinocyte induction in the healing process is unaffected. Although the connective tissue local immunity intensifies the galectin defense mechanism as well, this suggests that patients with an orofacial cleft have a prolonged immune response due to this defense factor. Notable differences in expression can be seen by comparing the milk group and the mixed dentition group patient muscle tissue, where the milk dentition palate shows a more elevated factor expressed in comparison to the mixed dentition palate.

Our knowledge of the cleft lip and or palate, especially its secreted local defense factors and their role in inflammation, is limited, and it requires more in-depth research. Lack of control in comparison to the patient group could present one of our most notable limitations; this would be explained by the fact that control samples are taken from healthy individuals, and are therefore more difficult to acquire. Immunohistochemistry was used to study our defense factor; however, different testing methods, such as ELISA, could prove to be valuable in comparing the levels of factor concentration, and how it affects the different types of the tissue. A partial limitation of the research could be that only one type of galectin was researched as other, more extensively studied factors (Gal-1,3,9), have been proven to have similar effects in other types of tissue [29]. In different eosinophilic diseases, Gal-10 expression levels have been connected to the level of inflammation involving clinical significance, which marks a future research topic [32]. Furthermore, as there have been research projects focusing on both milk and mixed dentition but searching for other different local defense factors, such as Human beta-defensin 2,3,4, Cathelicidin 37 and Interleukin-10, it could prove to be valuable to study a potential synergy of these factors with galectin-10 [12].

## Conclusions

The near absence of Gal-10 in the healthy palate, coupled with its increased expression in the palatal epithelium from the milk to the mixed dentition age in cleft-affected children suggests a possible role for this factor in providing local defense and epithelial barrier functions.

Additionally, the palatal muscle tissue is not the primary site for Gal-10 expression, either in healthy, or in cleft condition-affected individuals.

The sporadic and insignificant presence of Gal-10 in the palatal connective tissue of healthy individuals with milk dentition and in cleft-affected individuals with mixed dentition indicates individual variations in the palatal tissue that are not dependent on the specific disease.
